# Prognostic value of preoperative plasma fibrinogen level and platelet-to-lymphocyte ratio (F-PLR) in patients with localized upper tract urothelial carcinoma

**DOI:** 10.18632/oncotarget.13611

**Published:** 2016-11-25

**Authors:** Jiwei Huang, Yichu Yuan, Yanqing Wang, Jin Zhang, Wen Kong, Haige Chen, Yonghui Chen, Yiran Huang

**Affiliations:** ^1^ Department of Urology, Ren Ji Hospital, School of Medicine, Shanghai Jiao Tong University, Shanghai, China

**Keywords:** upper tract, urothelial carcinoma, fibrinogen, platelet-to-lymphocyte ratio, prognosis

## Abstract

**Purpose:**

Hemostatic factors is thought to have a potentially significant role in progression and metastasis of malignant tumors. We investigated the prognostic value of preoperative plasma fibrinogen level and platelet-to-lymphocyte ratio (PLR) in localized upper tract urothelial carcinoma (UTUC).

**Materials and Methods:**

A total of 481 patients who underwent radical nephroureterectomy for localized UTUC (pTa-4N0M0) were identified between January 2002 and June 2013. Patients were assigned a F-PLR score of 0, 1, or 2 based upon the presence of elevated plasma fibrinogen level, an elevated PLR, or both. The association between F-PLR score and clinicopathological variables was analysed.

**Results:**

The optimal cut-off value of plasma fibrinogen and PLR for overall survival stratification was determined to be 4.22 and 241.2. Kaplan–Meier analysis revealed significant differences in cancer specific survival (CSS) and overall survival (OS) among patients with F–PLR scores of 0, 1 and 2. Multivariate analysis identified higher F–PLR score as an independent risk factor for CSS (P < 0.001) and OS (P < 0.001). The estimated c-index of the multivariate model for CSS and OS increased from 0.772 and 0.756 to 0.799 and 0.784 when F–PLR score added, which was higher than fibrinogen level, PLR or neutrophil-to-lymphocyte ratio added.

**Conclusions:**

Preoperative F-PLR score is a negative independent prognostic factor for survival outcomes in patients with localized upper tract urothelial carcinoma. Preoperative F-PLR score may become a useful biomarker, particularly because of its low associated cost and easy accessibility.

## INTRODUCTION

Upper tract urothelial carcinoma (UTUC) is a relatively rare but aggressive malignant disease that accounts for only 5% to 10% of all urothelial carcinomas [1]. The natural history and oncogenicity of UTUC appear to be rather different from that of urothelial carcinoma of the bladder since around 50% of UTUCs are muscular invasive at diagnosis compared to only 15% of urothelial carcinomas of the bladder [2–3]. Even after standard surgery of radical nephroureterectomy (RNU) in patients with UTUC, the prognosis for localized UTUC patients still remains poor. Currently, 5-year cancer specific survivals (CSS) ranging between 50% and 80% have been reported [4–5]. Therefore, it is crucial to identify new risk factors, which would allow for better predicting individual surgical outcomes in localized UTUC patients who might benefit from individual patient treatment choices, such as neoadjuvant chemotherapy.

Cancer is associated with hypercoagulopathy and increased risk of thrombosis [6]. Growing evidences have demonstrated a significant role of hemostatic factors in the development of human cancer and metastasis [6–8]. Thrombosis, inflammation, and cancer are interrelated, and circulating blood platelets are one cellular element common to each process [8]. In the other hand, a number of procoagulant and fibrinolytic factors have been found to be overexpressed in the tumor [9]. Recent studies have proved that elevated plasma fibrinogen levels and platelet-to-lymphocyte ratio (PLR) are associated with worse clinical outcome in various human cancers [10–13]; however, there is no consensus as to which is a more reliable marker predicting the prognosis of cancer patients.

Thus, this study was designed to incorporate both markers into what we referred to as the F-PLR (fibrinogen and platelet-to-lymphocyte count ratio) score. Then, we investigate the prognostic role of the F-PLR score whether it could present a better predictive value for clinical outcome in patients with localized UTUC comparing with fibrinogen level, PLR or neutrophil-to-lymphocyte ratio (NLR).

## RESULTS

### Clinical characteristics

Our final cohort included 311 men (64.7%) and 170 women (35.3%). Patients’ mean age at surgery was 65.8±11.1 years (range, 30-89) (Table 1). Open RUN was performed in 318 patients (66.1%), while the remaining 163 patients (33.9%) underwent laparoscopic RUN, respectively. Among all patients, 232 (48.2%) had tumor in the renal pelvis, 160 (33.3%) had tumor in the ureter and 89 (18.5%) had multifocal lesions. Pathological T stage was pTa-1 in 248 cases (51.6%), pT2 in 76 (15.8%), pT3 in 142 (29.5%) and pT4 in 15 (3.1%). Lymphadenectomy was done in 107 patients (22.2%) who presented with clinically enlarged lymph nodes. 96 patients (20%) received adjuvant chemotherapy (AC) in this study.

**Table 1:** Clinical and pathological characteristics of 481 UTUC patients stratified according to F-PLR score

Variables	N (n=,%)	F-PLR score			P-value
		0(n=,%)	1(n=,%)	2(n=,%)	
Patients,n(%)	481(100.0)	333(69.2)	136(28.3)	12(2.5)	
Age (years)					0.108
<65	207(43.0)	153(45.9)	51(37.5)	3(25.0)	
≥65	274(57.0)	180(54.1)	85(62.5)	9(75.0)	
Gender,n(%)					0.599
Male	311(64.7)	220(66.1)	84(61.8)	7(58.3)	
Female	170(35.3)	113(33.9)	52(38.2)	5(41.7)	
Hypertension					0.100
No	327(68.0)	225(67.6)	97(71.3)	5(41.7)	
Yes	154(32.0)	108(32.4)	39(28.7)	7(58.3)	
Diabetes mellitus					0.023
No	417(86.7)	293(88.0)	117(86.0)	7(58.3)	
Yes	64(13.3)	40(12.0)	19(14.0)	5(41.7)	
Multifocality					0.050
No	392(81.5)	280(84.1)	104(76.5)	8(66.7)	
Yes	89(18.5)	53(15.9)	32(23.5)	4(33.3)	
T stage					<0.001
Ta-1	248(51.6)	199(59.8)	44(32.4)	5(41.7)	
T2-4	233(48.4)	134(40.2)	92(67.6)	7(58.3)	
N stage					0.044
N0/x	455(94.6)	320(96.1)	125(91.9)	10(83.3)	
N1-3	26(5.4)	13(3.9)	11(8.1)	2(16.7)	
WHO Grade					0.031
Low	163(33.9)	125(37.5)	36(26.5)	2(16.7)	
High	318(66.1)	208(62.5)	100(73.5)	10(83.3)	
LVI					<0.001
No	405(84.2)	295(88.6)	104(76.5)	6(50.0)	
Yes	76(15.8)	38(11.4)	32(23.5)	6(50.0)	
Adjuvant Chemotherapy					0.840
No	385(80.0)	266(79.9)	110(80.9)	9(75.0)	
Yes	96(20.0)	67(20.1)	26(19.1)	3(25.0)	

### Cut-off determination of fibrinogen, PLR and NLR

**Figure 1:**
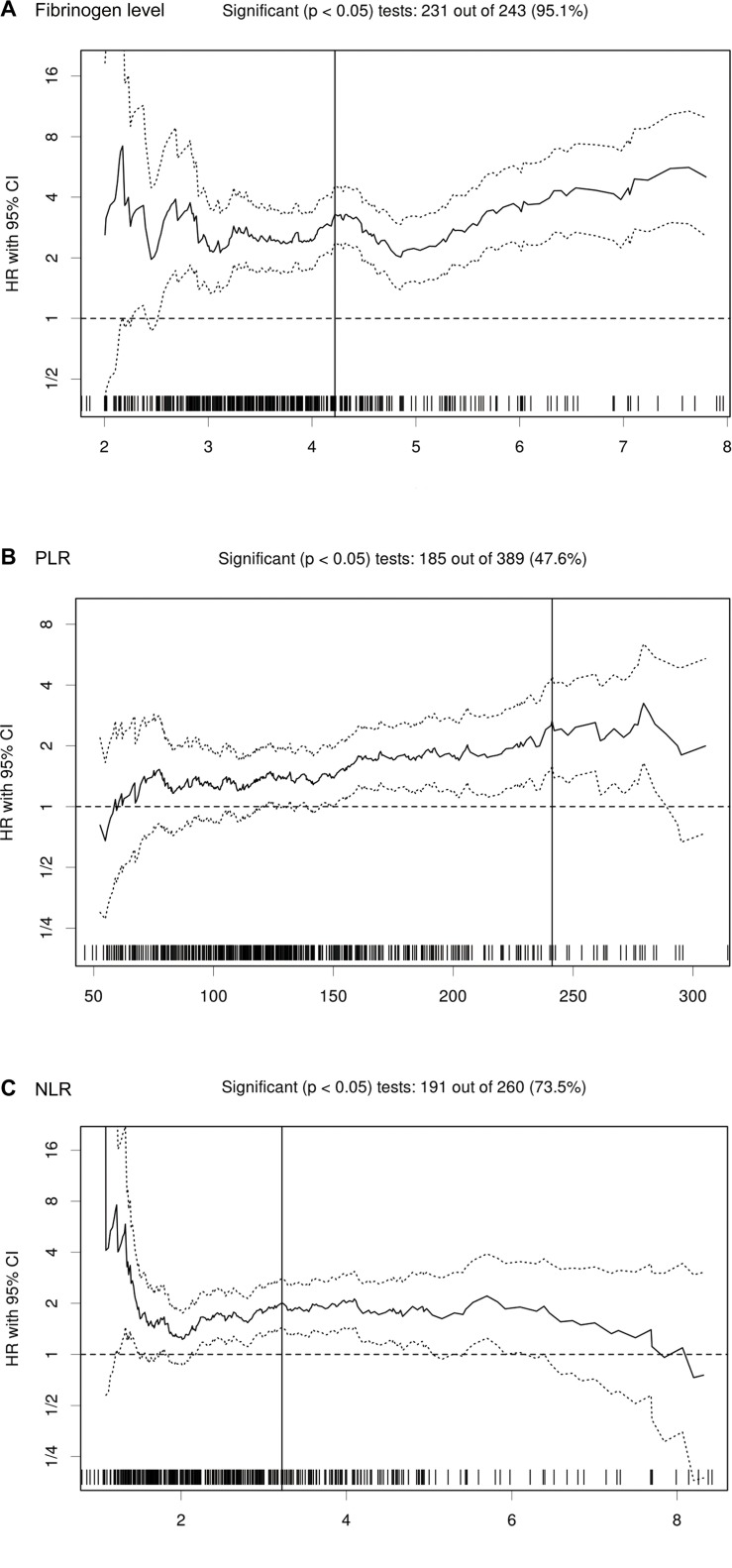
HR for OS in dependence of cut-off point for fibrinogen level A., PLR B. and NLR C. in UTUC patients The vertical line designates the optimal cut-off point with the most significant (log-rank test) split. The plots were generated using the biostatistical tool Cut-off Finder.

Using the biostatistical tool Cutoff Finder, we found that a wide range of cutoff points for fibrinogen (231 out of 243 tests, 95.1 %), PLR (185 out of 389 tests, 47.6 %), and NLR (191 out of 260 tests, 73.5 %) were significant (Figure 1). The optimal cut-off point of fibrinogen, PLR and NLR for the stratification of overall survival (OS) in UTUC was determined to be 4.22, 241.2 and 3.22.

### Association with clinical and pathologic characteristics

Patients were categorized into 3 groups, of which 333 (69.2%) patients had a F-PLR score of 0, 136 (28.3%) had a F-PLR score of 1 and 12 (2.5%) had a F-PLR score of 2. There were significant differences between F-PLR score of 0,1 and 2 in terms of diabetes mellitus (p = 0.023), pT stage (p < 0.001), pN stage (p = 0.044), grade (p = 0.031) and lymphovascular invasion (LVI) (p < 0.001) (Table 1).

### Association with prognosis of UTUC

**Figure 2:**
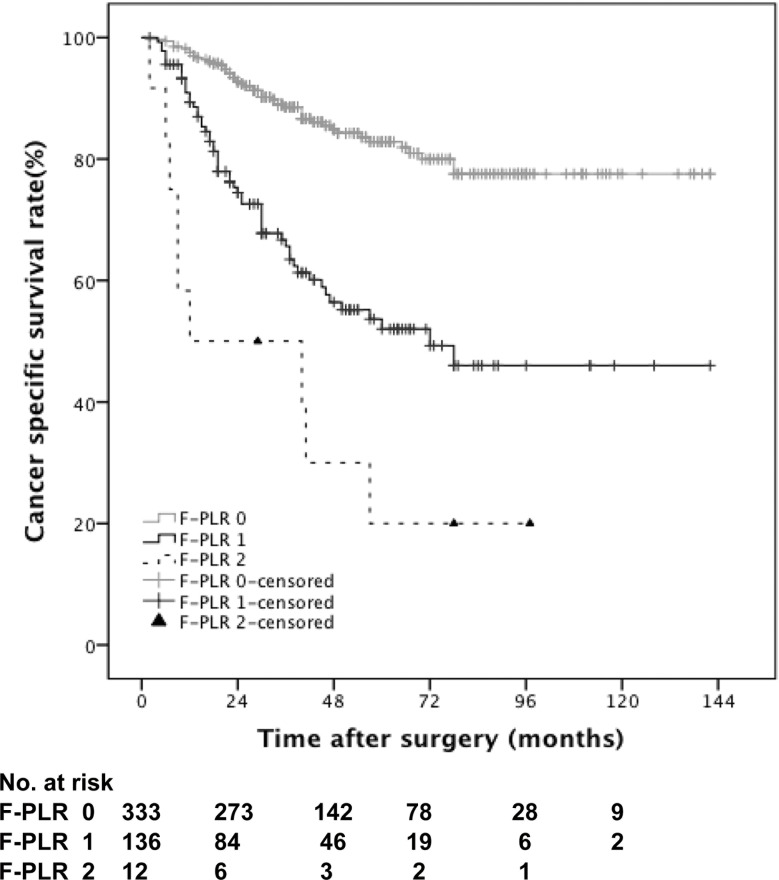
Kaplan-Meier curves predicting CSS by preoperative F-PLR score

**Figure 3:**
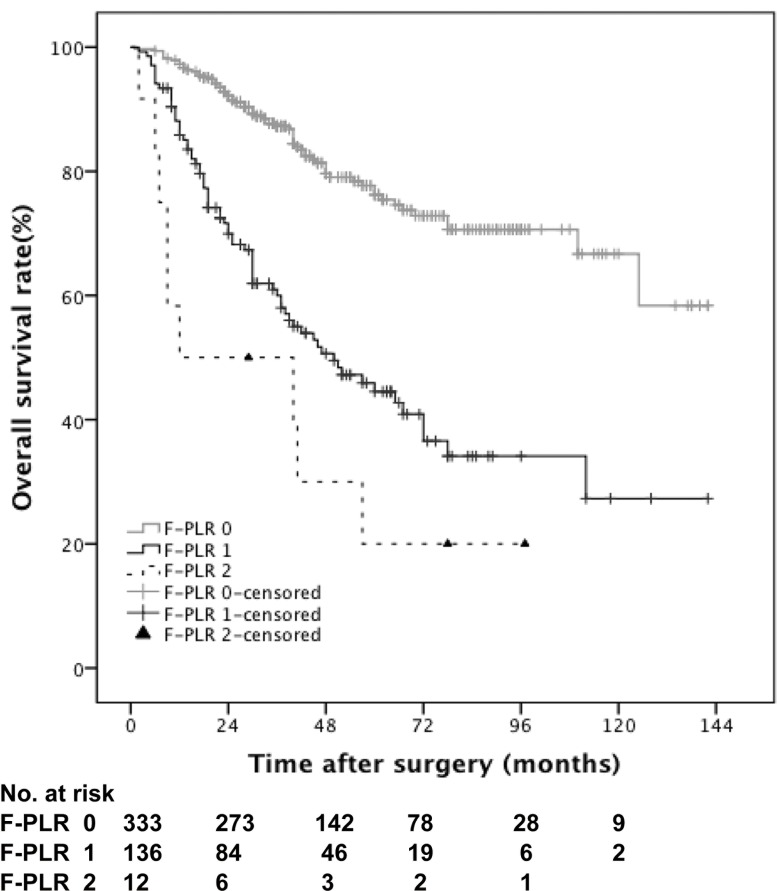
Kaplan-Meier curves predicting OS by preoperative F-PLR score

At a median follow-up period of 40 months (interquartile range, 24.0-64.0), 145 (30.1%) of 481 patients experienced death, including 112 (23.3%) died from UTUC. Higher F-PLR score was associated significantly with worse CSS outcomes (p < 0.001, score 2/0; p < 0.001, score 1/0; p = 0.012, score 2/1) (Figure 2), while similar results were observed with poorer OS outcomes (p < 0.001, score 2/0; p < 0.001, score 1/0; p = 0.080, score 2/1) (Figure 3). The 5-year CSS rate was 82.8% in patients with F-PLR score of 0, 52% in patients with F-PLR score of 1 and 20% in patients with F-PLR score of 2 (Figure 2). Meanwhile the 5-year OS rate was 76.2% in patients with F-PLR score of 0, 44.5% in patients with F-PLR score of 1 and 20% in patients with F-PLR score of 2 (Figure 3). In the subgroup of patients regardless of muscle invasion or not or any grade, higher F-PLR score predicted worse CSS and OS (Log-rank test, each p < 0.05) (Supplementary Figure 1 and 2).

Tables 2 and 3 showed the results of the stepwise Cox survival analyses for predicting the CSS and OS, respectively. Multivariable analysis showed that F-PLR score (hazard ratio (HR), 5.11, P<0.001, score 2/0; HR, 2.28, P<0.001, score 1/0) was an independent predictor of CSS, along with age greater than 65 (HR, 2.00; P<0.001), tumor multifocality (HR, 1.68; P=0.016), higher pT stage (HR, 2.79; P<0.001), higher grade (HR, 2.08; P=0.006) and LVI (HR, 1.59;P=0.032) (Table 2). As to OS, multivariable analysis also demonstrated that F-PLR score (HR, 3.78, P<0.001, score 2/0; HR, 2.39, P<0.001, score 1/0) was an independent predictor of OS, along with age greater than 65 (HR, 2.38; P<0.001), tumor multifocality (HR, 1.49; P=0.040), higher pT stage (HR, 2.17; P<0.001), higher pN stage (HR, 1.71; P=0.070), higher grade (HR, 1.65; P=0.019) and LVI (HR, 1.43;P=0.071) (Table 3).

**Table 2:** Univariable and multivariable Cox regression models to predict CSS in 481 patients treated with radical nephroureterectomy for UTUC

Variables	CSS Multivariable		
	p Value	HR (95%CI)	p Value
Age (years)	<0.001		
<65		Reference	
≥65		2.00(1.31-3.03)	0.001
Gender	0.540		
Male			
Female			
Hypertension	0.950		
No			
Yes			
Diabetes mellitus	0.023	-	
No			
Yes			
Multifocality	<0.001		
No		Reference	
Yes		1.68(1.10-2.55)	0.016
pTNM stage			
T stage	<0.001		
Ta-1		Reference	
T2-4		2.79(1.72-4.53)	<0.001
N stage	<0.001	-	
Nx/0			
N1-3			
WHO Grade	<0.001		
Low		Reference	
High		2.08(1.23-3.52)	0.006
LVI	<0.001		
No		Reference	
Yes		1.59(1.04-2.42)	0.032
Adjuvant Chemotherapy	0.144		
No			
Yes			
F-PLR score	<0.001		
0		Reference	
1		2.28(1.53-3.41)	<0.001
2		5.11(2.44-10.72)	<0.001

**Table 3:** Univariable and multivariable Cox regression models to predict OS in 481 patients treated with radical nephroureterectomy for UTUC

Variables	CSS Multivariable		
	p Value	HR (95%CI)	p Value
Age (years)	<0.001		
<65		Reference	
≥65		2.38(1.62-3.50)	<0.001
Gender	0.987		
Male			
Female			
Hypertension	0.784		
No			
Yes			
Diabetes mellitus	0.004	-	
No			
Yes			
Multifocality	0.001		
No		Reference	
Yes		1.49(1.02-2.19)	0.040
pTNM stage			
T stage	<0.001		
Ta-1		Reference	
T2-4		2.17(1.45-3.24)	<0.001
N stage	<0.001		
Nx/0		Reference	
N1-3		1.71(0.96-3.04)	0.070
WHO Grade	<0.001		
Low		Reference	
High		1.65(1.09-2.52)	0.019
LVI	<0.001		
No		Reference	
Yes		1.43(0.97-2.09)	0.071
Adjuvant Chemotherapy	0.665		
No			
Yes			
F-PLR score	<0.001		
0		Reference	
1		2.39(1.68-3.39)	<0.001
2		3.78(1.81-7.89)	<0.001

The predictive accuracy was calculated with and without the inclusion of fibrinogen, PLR, NLR and F-PLR score. In the base model, including the traditional predictor variables of age, tumor multifocality, pT stage, pN stage, higher grade and LVI, predictive accuracy for CSS was 77.2%; with the addition of fibrinogen, predictive accuracy was 79.4%; with the addition of PLR, predictive accuracy was 77.5%, with the addition of NLR, predictive accuracy was 77.9%, with the addition of F-PLR score, predictive accuracy was 79.9% (Table 4). As to OS, we observed similar results, in the base model, including the traditional predictor variables of age, tumor multifocality, pT stage, pN stage, higher grade and LVI, predictive accuracy was 75.6%; with the addition of fibrinogen, predictive accuracy was 77.6%; with the addition of PLR, predictive accuracy was 75.8%, with the addition of NLR, predictive accuracy was 75.8%, with the addition of F-NLR score, predictive accuracy was 78.4% (Table 5).

**Table 4:** Multivariable model of possible independent prognostic variables for CSS in UTUC patients

Co-Variable	Multivariable	*P* Value	Multivariable	*P* Value	Multivariable	*P* Value	Multivariable	*P* Value	Multivariable	*P* Value
	HR(95%CI)		HR(95%CI)		HR(95%CI)		HR(95%CI)		HR(95%CI)	
Age (≥65 vs<65 years)	2.07(1.37-3.14)	0.001	1.99(1.31-3.02)	0.001	2.04(1.34-3.09)	0.001	2.03(1.34-3.08)	0.001	1.96(1.29-2.99)	0.002
Multifocality(yes vs no)	1.95(1.29-2.95)	0.002	1.78(1.18-2.70)	0.007	1.85(1.21-2.82)	0.005	1.79(1.18-2.72)	0.006	1.66(1.09-2.53)	0.018
pT (2-4vs a,1)	3.19(2.00-5.12)	<0.001	2.68(1.64-4.36)	<0.001	3.16(1.97-5.06)	<0.001	3.01(1.87-4.83)	<0.001	2.75(1.70-4.45)	<0.001
pN(1-3 vs x/0)	1.83(0.99-3.39)	0.054	1.57(0.84-2.93)	0.16	1.72(0.92-3.21)	0.089	1.67(0.90-3.10)	0.108	1.39(0.73-2.65)	0.31
Grade (High vs Low)	2.12(1.27-3.56)	0.004	2.02(1.19-3.43)	0.009	2.15(1.28-3.60)	0.004	2.21(1.32-3.71)	0.003	2.08(1.23-3.51)	0.006
LVI (yes vs no)	1.72(1.14-2.59)	0.01	1.72(1.14-2.59)	0.01	1.64(1.08-2.49)	0.021	1.72(1.14-2.59)	0.01	1.54(1.01-2.34)	0.045
Fibrinogen level (>4.22 vs ≤4.22)			2.56(1.75-3.76)	<0.001						
PLR (>241.2 vs ≤241.2)					1.53(0.84-2.79)	0.161				
NLR (>3.22 vs ≤3.22)							1.73(1.18-2.54)	0.005		
F-RLR score (2 vs 1 vs 0)									2.21(1.61-3.03)	<0.001
Predictive Accuracy (%)	77.2		79.4		77.5		77.9		79.9	

**Table 5:** Multivariable model of possible independent prognostic variables for OS in UTUC patients

Co-Variable	Multivariable	*P* Value	Multivariable	*P* Value	Multivariable	*P* Value	Multivariable	*P* Value	Multivariable	*P* Value
	HR(95%CI)		HR(95%CI)		HR(95%CI)		HR(95%CI)		HR(95%CI)	
Age (≥65 vs<65 years)	2.53(1.73-3.71)	<0.001	2.44(1.66-3.58)	<0.001	2.49(1.69-3.65)	<0.001	2.49(1.70-3.65)	<0.001	2.39(1.63-3.51)	<0.001
Multifocality(yes vs no)	1.76(1.21-2.57)	0.003	1.61(1.10-2.35)	0.014	1.65(1.12-2.43)	0.011	1.62(1.11-2.38)	0.013	1.50(1.03-2.21)	0.037
pT (2-4vs a,1)	2.52(1.71-3.71)	<0.001	2.18(1.46-3.26)	<0.001	2.49(1.69-3.66)	<0.001	2.40(1.63-3.54)	<0.001	2.23(1.50-3.31)	<0.001
pN(1-3 vs x/0)	2.09(1.20-3.64)	0.009	1.81(1.03-3.18)	0.039	1.96(1.12-3.44)	0.019	1.96(1.12-3.42)	0.018	1.63(0.92-2.90)	0.095
Grade (High vs Low)	1.71(1.13-2.59)	0.011	1.63(1.07-2.50)	0.023	1.73(1.15-2.62)	0.009	1.77(1.17-2.67)	0.007	1.68(1.10-2.55)	0.016
LVI (yes vs no)	1.51(1.04-2.20)	0.032	1.53(1.05-2.23)	0.027	1.43(0.98-2.11)	0.066	1.51(1.04-2.20)	0.032	1.39(0.94-2.03)	0.096
Fibrinogen level (>4.22 vs ≤4.22)			2.45(1.75-3.43)	<0.001						
PLR (>241.2 vs ≤241.2)					1.61(0.94-2.76)	0.082				
NLR (>3.22 vs ≤3.22)							1.58(1.12-2.22)	0.009		
F-RLR score (2 vs 1 vs 0)									2.16(1.64-2.84)	<0.001
Predictive Accuracy (%)	75.6		77.6		75.8		75.9		78.4	

## DISCUSSION

Despite recent progress in the identification of genetic and molecular alterations in UTUC, the routine prognostic risk assessment of UTUC patients currently still relies on traditional well established clinicopathological prognostic factors, including age, clinical tumor stage, tumor grade, tumor multifocality, and LVI [14–16]. The predictive accuracy of this traditional prognostic model need be further improved by the incorporation of novel prognostic biomarkers, which might benefit for individual patent treatment choices, such as neoadjuvant chemotherapy.

In the present study, we investigated F-PLR score, fibrinogen level, PLR, NLR and other standard prognostic factors in 481 patients undergoing RNU for localized UTUC. Our study showed that in addition to other well-established prognostic factors, F-PLR score was an independent predictor of CSS and OS. To the best of our knowledge, our analysis was the first study to incorporate fibrinogen and PLR together to evaluate whether the combination of these two hemostatic factors could present a better predictive value for cancer patients’ survival outcome. Our subgroup analysis also showed that higher F-PLR score could predict poorer prognosis in the subgroup of UTUC patients with any tumor grade or muscle invasion or not. In the multivariate model analysis, our new model combine F-PLR score and traditional clinicopathological prognostic factors (age, clinical tumor stage, tumor grade, tumor multifocality, and LVI together showed better predictive accuracy than the base model, base model with addition of fibrinogen level, PLR or NLR.

Cancer is associated with increased risk of thrombosis and coagulation pathway activation. More and more evidences have recognized that hemostatic variables, particularly platelet counts and plasma fibrinogen levels, have prognostic significance in patients with cancer [10–13, 17]. Platelets and fibrinogen together actively plays an important role in tumor growth, invasion and hematogenous metastasis by promoting tumour neovascularization and by supporting the sustained adhesion of tumour cells [8, 18–19]. Several studies showed an important link between hemostatic factors and innate immunity and indicate that one mechanism by which the platelet-fibrin(ogen) axis contributes to metastatic potential is by impeding natural killer cell elimination of tumor cells [7]. In various types of cancer, thrombocytosis and PLR were proved to have a close relationship with survival outcomes [10–11, 17]. Recently, a meta-analysis of 12 studies including 8735 renal cell carcinoma patients showed thrombocytosis had a significant influence of 5-year OS and CSS in patients with localized renal cell carcinoma [17]. You et al. demonstrated that elevated PLR level could significantly increase the rate of overall survival outcome in colorectal cancer patients after surgery [11]. On the other hand, Feng et al. showed that fibrinogen level was found to be an independent predictor for OS in the high-grade serous ovarian cancer patients [10]. Recently, the elevated plasma fibrinogen level was also proved to be an independent prognostic factor of CSS and OS in UTUC patients [12–13]. So we hypothesized that the F-PLR sore which combined these two biomarker together may be better predictor for prognosis of UTUC.

In our study, we found that in the present cohort of patients with UTUC, those who had higher F-PLR score were more likely to have higher T, N stage, tumor grade and higher rate of LVI comparing with those with low F-PLR score. These differences between patients with different F-PLR scores in terms of tumor characteristics may partly explain why the patients with higher F-PLR score in our cohort had more aggressive disease.

Preoperative NLR was a new biomarker of systemic inflammatory response, which recently had been proved to be an independent predictor for prognosis of UTUC in several large cohort studies [5, 20]. In our study, we also want to determine whether F-PLR sore was a better predictor for survival outcome of UTUC comparing with NLR. The results showed that adding F-PLR was able to raise the predictive accuracy in this cohort of UTUC patients regarding to CSS and OS even comparing with adding fibrinogen level, PLR or NLR. The base model of CSS prediction, which included the traditional predictor variables of TNM stage, age, tumor multifocality, grade and LVI, was of a predictive accuracy (77.2%), which could be further improved by the addition of fibrinogen level (79.4%), PLR (77.5%), NLR (77.9%) or F-PLR (79.9%). As to OS prediction, the predictive accuracy of base model was 75.6%, while the predictive accuracy increased to 77.6%, 75.8%, 75.9% or 78.4% by including the fibrinogen level, PLR, NLR or F-PLR score. Considering F-PLR score that is widely available and relatively easy to assess even before surgery, they may become attractive variables for patients counseling and individual patient treatment choices, such as neoadjuvant chemotherapy and lymph node dissection, because of the poor patient prognosis in UTUC.

Limitations of this study include that the retrospective nature of the data collection and study completed at a single high-volume center. Prospective studies with a larger population are warranted so as to determine the accurate prognostic role of F-PLR score in patients with UTUC.

## MATERIALS AND METHODS

### Patients

After obtaining institutional review board approval, we retrospectively reviewed 526 consecutive patients with localized UTUC (Ta-4N0/+M0) underwent RNU at the department of urology at our institution between January 2002 and June 2013. The following patients were excluded: 1) patients lost to follow-up within 3 months; 2) patients without data on preoperative preoperative plasma fibrinogen levels, platelet and lymphocyte counts; 3) patients with an active infection, a haematological disorder, or acute or chronic inflammatory and/or autoimmune disease; 4) patients who had undergone previous steroid therapy; 5) patients with concomitant carcinoma invading bladder muscle; and 6) patients who underwent cisplatin-based neoadjuvant chemotherapy. Finally there were 481 remaining patients who were included in the analyses.

### Clinical and pathologic evaluation

Clinical features including patient age, gender, hypertension, diabetes mellitus, pathological diagnosis and therapeutic information were obtained from the medical records. Dissection of regional lymph nodes was performed in patients with lymph nodes enlarged in preoperative imaging. Pathological T and N stages were uniformly adjusted according to the 2009 TNM classification system [21] and M stage was assigned clinically before surgery. Tumor grade was assessed according to Tumor grading was assessed according to the 1998 WHO consensus classification [22]. We defined multifocal tumors as follows: the synchronous presence of two or more pathologically confirmed tumors in any location within the upper urinary tract [23]. Blood samples taken within 1 week before surgery were tested for plasma fibrinogen and for neutrophil, lymphocyte and platelet counts. AC would be offered as an option to patients with muscle invasive disease, lymph node positive disease, or pT1 disease with LVI, high grade disease or tumor multifocality after surgery. The patient would make a decision after the benefits and side effects of postoperative AC were explained by the treating urologist and oncologist together. The chemotherapy patients were administered 1000 mg/m2 gemcitabine on day 1, and 8 and 70 mg/m2 cisplatin on day 2 for the GC regimen. Cisplatin was replaced by carboplatin if the glomerular filtration rate was less than 40 ml/minute/1.73 m^2^. Four to 6 cycles of chemotherapy were planned according to patient status.

### Follow-up

Patients were assessed by urine cytology and cystoscopy 3 months and every 6 months for 3 years after RNU and every 12 months thereafter. Computed tomography and/or magnetic resonance imaging were also performed every 6 months for 3 years and annually thereafter. CSS was defined as the time in months from the date of surgery to cancer related death. OS was defined as the time in months from the date of surgery to patient death from any cause. Cause of death was determined by treating physicians and institutional cancer registries, by chart review corroborated by death certificates, or by death certificates alone. All patients who were coded as dead of cancer had previous disease progression.

### Diagnostic criteria

The PLR was defined as the absolute platelet count divided by the absolute lymphocyte count. Similarly, the NLR was defined as the absolute neutrophil count divided by the absolute lymphocyte count. The optimal cut-off value of fibrinogen, PLR and NLR was determined using a minimum p value approach for OS. Patients were assigned a F-PLR score of 0, 1, or 2 based on the presence of increased fibrinogen level, an elevated PLR, or both, as follows: patients with both elevated fibrinogen and PLR were assigned a score of 2, and patients with either or neither were assigned a score of 1 or 0, respectively.

### Statistical analysis

For continuous variables, the Student t test was used for the variables reported as mean (±standard deviation) or median (range or interquartile range), for categorical variables, the chi-square and Fisher's exact tests were used. Unlike most of the studies used median or mean value as cut-off and we chose minimum p value approach for OS to determined our optimal cut-off value of fibrinogen, PLR and NLR by minimum p value approach for OS using a R software-engineered, web-based system designed by Budczies et al. (http://molpath.charite.de/cutoff/) [24]. CSS and OS were calculated using the Kaplan-Meier method and compared by the log rank test, and subgroup analyses were taken according to grade or presence of muscle invasion. Backward stepwise multivariate Cox proportional analysis was performed to determine the influence of F-PLR and other clinical and pathologic variables on CSS and OS. HR and 95% confidence interval (CI) were computed. The Harrell c-index was used to assess the predictive accuracy of the model on multivariate analysis and for comparison after supplementation by fibrinogen, PLR, NLR and F-PLR score. All p values reported are two-sided, and p<0.05 was considered statistically significant. The statistical software SPSS, v21.0 (IBM Corp., Armonk, NY, USA) was applied to all the analyses in this study.

## SUPPLEMENTARY MATERIALS FIGURES


