# Supplementary material for: Prognostic value of preoperative plasma fibrinogen level and platelet-to-lymphocyte ratio (F-PLR) in patients with localized upper tract urothelial carcinoma
Source: Oncotarget. 2016 Nov 25;8(22):36761–71. doi: 10.18632/oncotarget.13611 (PMC5482695; doi:10.18632/oncotarget.13611)
Supplement: Supplementary file 1 [file oncotarget-08-36761-s001.pdf]

Prognostic value of preoperative plasma fibrinogen level and platelet-to-lymphocyte ratio (F-PLR) in patients with localized upper tract urothelial carcinoma

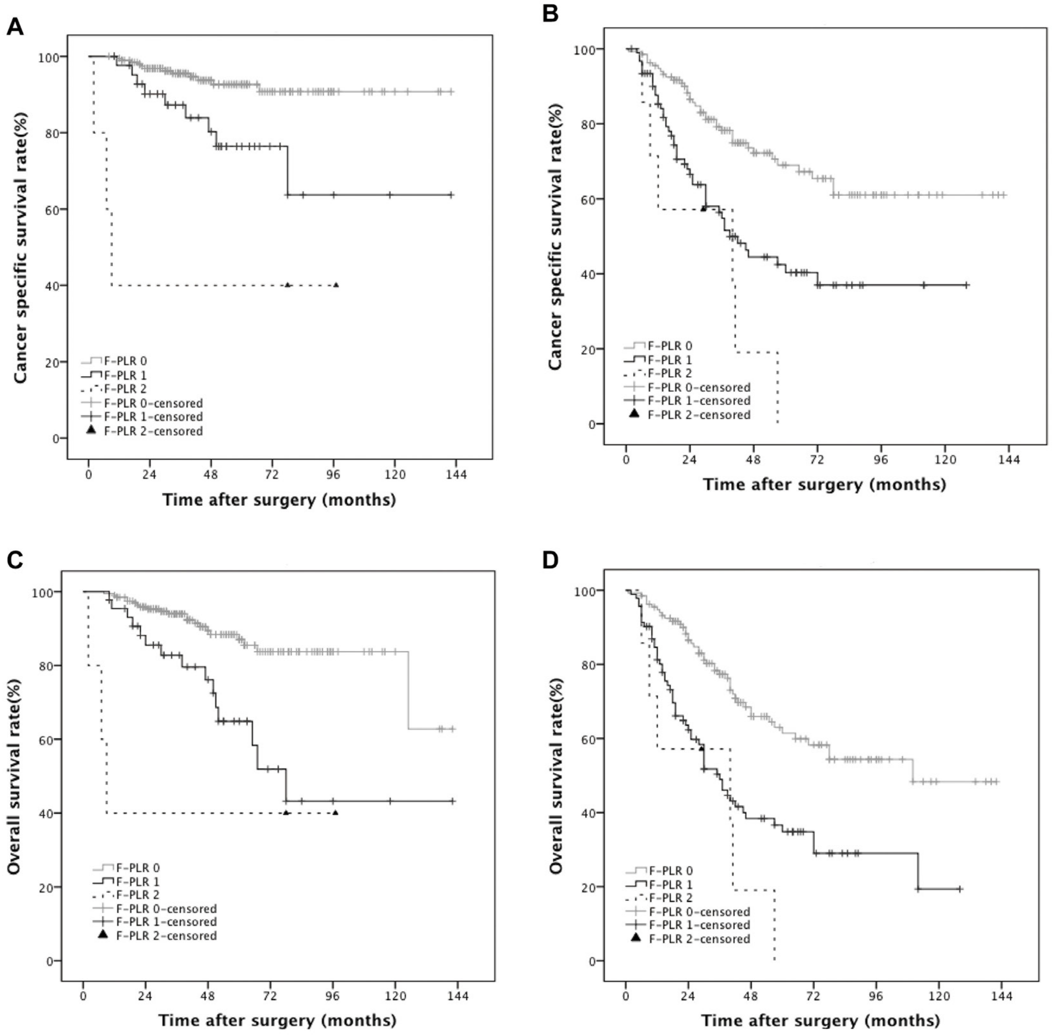

Supplementary Figure 1: Kaplan-Meier curves stratified by F-PLR score predicting CSS in localized UTUC patients with pTa-1 stage A. and pT2-4 stage B.; predicting OS in localized UTUC patients with pTa-1 stage C. and pT2-4 stage D.

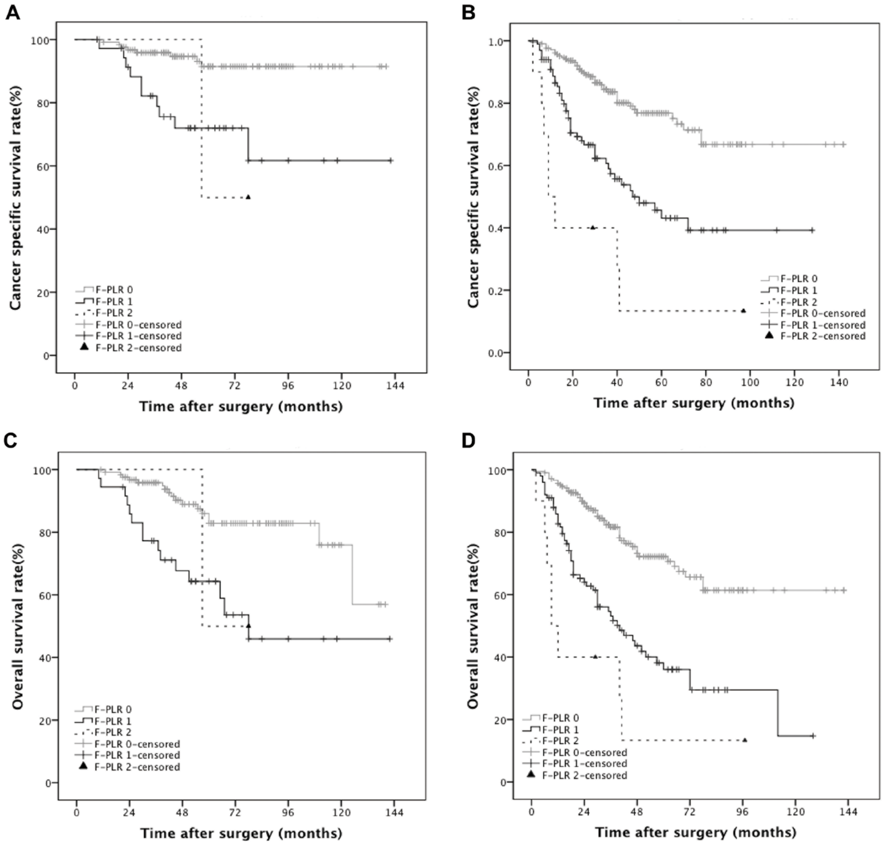

Supplementary Figure 2: Kaplan-Meier curves stratified by F-PLR score predicting CSS in localized UTUC patients with low grade A. and high grade B.; predicting OS in localized UTUC patients with low grade C. and high grade D.
